# Prognostic impact of serum CYFRA 21–1 in patients with advanced lung adenocarcinoma: a retrospective study

**DOI:** 10.1186/1471-2407-13-354

**Published:** 2013-07-23

**Authors:** Akira Ono, Toshiaki Takahashi, Keita Mori, Hiroaki Akamatsu, Takehito Shukuya, Tetsuhiko Taira, Hirotsugu Kenmotsu, Tateaki Naito, Haruyasu Murakami, Takashi Nakajima, Masahiro Endo, Nobuyuki Yamamoto

**Affiliations:** 1 Division of Thoracic Oncology, Shizuoka Cancer Center, 1007, Shimonagakubo, Nagaizumi-cho, Sunto-gun, Shizuoka 411-8777, Japan; 2 Division of Diagnostic Pathology, Shizuoka Cancer Center, 1007, Shimonagakubo, Nagaizumi-cho, Sunto-gun, Shizuoka 411-8777, Japan; 3 Division of Diagnostic Radiology, Shizuoka Cancer Center, 1007, Shimonagakubo, Nagaizumi-cho, Sunto-gun, Shizuoka 411-8777, Japan; 4 Shizuoka Cancer Center, Clinical Trial Coordination Office, 1007, Shimonagakubo, Nagaizumi-cho, Sunto-gun, Shizuoka 411-8777, Japan

**Keywords:** Lung adenocarcinoma, Prognostic factor, CYFRA 21–1, CEA, EGFR mutation, Tumor heterogeneity, EGFR-TKI, Chemotherapy

## Abstract

**Background:**

Serum CYFRA 21–1 is one of the most important serum markers in the diagnosis of non-small cell lung cancer (NSCLC), especially squamous-cell carcinoma. However, it remains unknown whether pretreatment serum CYFRA 21–1 values (PCV) may also have prognostic implications in patients with advanced lung adenocarcinoma.

**Methods:**

We retrospectively reviewed the data of 284 patients (pts) who were diagnosed as having advanced lung adenocarcinoma and had received initial therapy.

**Results:**

Of the study subjects, 121 pts (43%) had activating epidermal growth factor receptor (EGFR) mutations (Mt+), while the remaining 163 pts (57%) had wild-type EGFR (Mt-). Univariate analysis identified gender (male/ female), ECOG performance status (PS) (0-1/ ≥2), PCV (<2.2 ng/ml/ ≥2.2 ng/ml), EGFR mutation status (Mt+/ Mt-), pretreatment serum CEA values (<5.0 ng/ml/ ≥5.0 ng/ml), smoking history (yes/ no) and EGFR-TKI treatment (yes/ no) as prognostic factors (p = .008, p < .0001, p < .0001, p < .0001, p = .036, p = .0012, p < .0001 respectively). Cox's multivariate regression analysis identified PCV < 2.2ng/ml as the only factor significantly associated with prolonged survival (p < .0001, hazard ratio: 0.43, 95% CI 0.31-0.59), after adjustments for PS (p < .0001), EGFR mutation status (p = .0069), date of start of initial therapy (p = .07), gender (p = .75), serum CEA level (p = .63), smoking history (p = .39) and EGFR-TKI treatment (p = .20). Furthermore, pts with Mt+ and PCV of <2.2 ng/ml had a more favorable prognosis than those with Mt+ and PCV of ≥2.2 ng/ml (MST: 67.0 vs. 21.0 months, p < .0001), and patients with Mt- and PCV of <2.2 ng/ml had a more favorable prognosis than those with Mt- and PCV of ≥2.2 ng/ml (MST: 24.1 vs. 10.2 months, p < .0001).

**Conclusion:**

PCV may be a potential independent prognostic factor in both Mt+ and Mt- patients with advanced lung adenocarcinoma.

## Background

Lung cancer is the leading cause of cancer death, and at present, there exists no cure of stage IV non-small cell lung cancer (NSCLC) [1]. Adenocarcinoma and squamous cell carcinoma are the most common histological subtypes of lung cancer and account for about 70% of all lung cancers [2]. The folate antagonist pemetrexed has been shown to exhibit efficacy against non-squamous cell lung cancers [3], and is currently used in combination with cisplatin as a standard treatment regimen for patients with non-squamous cell lung carcinoma. Chemotherapy with the angiogenesis inhibitor bevacizumab administered in combination with platinum agents has also been shown to exhibit favorable efficacy against non-squamous cell lung carcinoma [4,5]. Somatic gain-of-function mutations in exons encoding the EGFR tyrosine kinase domain have been identified in NSCLC [6,7]. Several previous studies have reported prolongation of the survival time in patients with EGFR-mutation-positive lung carcinomas treated with EGFR-tyrosine kinase inhibitors (TKIs) [8-11], therefore, EGFR-TKIs are widely used in medical practice. EGFR mutations occur more frequently in lung cancer patients who are Asians, females and non-smokers with the histological subtype of adenocarcinoma [12-14]. On the other hand, while there have also been scattered reports of EGFR mutations among cases of lung squamous-cell carcinoma [15-17], a recent report showed that there were no EGFR mutation-positive cases among lung cancer patients with pure squamous cell carcinoma [18,19].

CYFRA 21–1 is a fragment of cytokeratin (CK) 19. CKs, which are now called keratins, are the principal structural elements of the cytoskeleton (keratin filaments) of epithelial cells, including bronchial epithelial cells, and have been classified into 20 subtypes based on differences in the molecular mass and isoelectric point as determined by 2-dimensional electrophoresis [20,21]. CK types 1–8 are categorized as type I CKs, and CKs 9–20 as type II CKs. Microfilaments are heteropolymers formed from type I and type II keratins, and constitute the cytoskeleton [22]. CK19 is a soluble type I CK (acidic type), and has the lowest molecular mass (40 kDa) among the CKs. It is expressed in the unstratified or pseudostratified epithelium lining the bronchial tree [23], and been reported to be overexpressed in many lung cancer tissue specimens [24]. The CK expression patterns in tissues are well-maintained even during the process of transformation of the tissue from normal to tumor tissue [25]. Accelerated CK19 degradation occurs in neoplastically transformed epithelial cells as a result of increased protease activity of caspase 3, a regulator of the apoptosis cascade, and fragments are released into the blood. This results in an increase of the blood CYFRA 21–1 values, because CK19 fragments are recognized by two monoclonal antibodies [26].

Measurement of serum CYFRA 21–1 level is a useful auxiliary test in the diagnosis of NSCLC, and particularly high specificity of this test has been reported for the diagnosis of squamous cell carcinoma of the lung [27,28]. On the other hand, a meta-analysis also revealed that serum CYFRA 21–1 may be a useful prognostic factor in NSCLC patients [29]; analysis of the histological background in the aforementioned meta-analysis showed that non-adenocarcinoma accounted for the majority of cases of NSCLC (65%). There has also been a report suggesting that serum CYFRA 21–1 levels might serve as a prognostic factor in patients with recurrent NSCLC receiving 3^rd^-line or later gefitinib therapy [30]. Some studies have suggested the possible prognostic value of pretreatment serum CYFRA 21–1 values (PCV) in patients with surgically treated lung adenocarcinoma [31] and advanced NSCLC [32-34]. However, none of the studies suggesting serum CYFRA 21–1 as a prognostic factor in patients with untreated advanced lung adenocarcinoma has included the EGFR mutation status as a variable. Therefore, in the present study, we investigated the impact of serum CYFRA 21–1 on the prognosis of untreated advanced lung adenocarcinoma patients.

## Methods

### Patients

**Figure 1:**
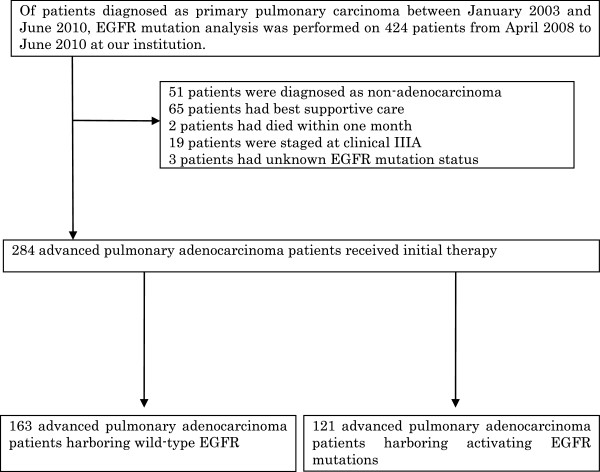
A flow-diagram of the patients included in the analysis.

Of patients diagnosed as having primary lung carcinoma between January 2003 and June 2010 at the Shizuoka Cancer Center, EGFR mutation analysis was performed on 424 patients from April 2008 to June 2010. Of these, 284 lung adenocarcinoma patients had received initial therapy, and we retrospectively reviewed the data of the 163 patients who were found to harbor wild-type EGFR and 121 patients who were found to harbor activating EGFR mutations (Figure 1). The following inclusion criteria were set for this study; patients with pathologically proven adenocarcinoma who had received initial therapy (including chemotherapy or chemoradiotherapy) and survived for more than one month; Eastern Cooperative Oncology Group performance status (ECOG PS) of 3 or less. The histological and cytological diagnoses were performed according to the WHO classification criteria [35]. The study was conducted with the approval of the Shizuoka cancer center Institutional Review Board #1 (HHS IRB registration number; IRB00006744).

We outsourced some of the clinical laboratory tests, such as measurement of the tumor markers and EGFR mutation analysis. Serum CYFRA 21–1 and serum CEA concentrations were measured at the baseline, before the initial therapy. The serum CYFRA 21–1 concentration was measured using a Lumipulse Presto® kit (FUJIREBIO Inc, Tokyo, Japan), based on a CLEIA (chemiluminescent enzyme immunoassay) method, while the serum CEA concentrations were measured using an ARCHITECT® kit (Abbott Japan, Tokyo, Japan). EGFR mutation analysis was performed by fragment analysis using polymerase chain reaction (PCR) and the cycleave real-time quantitative PCR technique (SRL Inc, Tokyo, Japan).

The reported upper limit of normal for the diagnosis of NSCLC and upper limit of the percentiles for healthy individuals of serum CYFRA 21–1 as measured by EIA are 3.5 ng/ml and 2.8 ng/ml, respectively [36]. In contrast, the reported upper limit of the percentiles for healthy individuals of serum CYFRA 21–1 measured by the CLEIA method is 1.6 ng/ml [37], a lower value as compared to that set for measurement by the EIA method. Therefore, for our study, we set the cutoff value for CYFRA 21–1 at 2.2 ng/ml, based on the mean value for healthy subjects + 3SD [37], a lower value as compared to that set for measurement by the EIA method. The cutoff value for serum CEA was set at 5.0 ng/ml, which is the upper limit of normal.

A standard evaluation of the patients, including assessment of the medical history, physical examination and routine laboratory tests, was performed before each treatment. All patients were staged based on the International Association for the Study of Lung Cancer (IASLC) TNM (tumor-node-metastasis) classification, 7^th^ edition [38].

### Statistical methods

There were no missing data in our study. Survival was estimated using the Kaplan-Meier method. Overall survival was measured from the date of the first course of the initial therapy to the date of death or that of the last follow-up examination. A log-rank test was performed to evaluate the significance of differences in the overall survival among the groups. P values < 0.05 were considered to be indicative of statistical significance. A multivariate analysis using the Cox proportional hazards model was used to establish the association between the clinical variables and survival. All statistical analyses were carried out using SPSS, version 11.0 for Windows (SPSS Inc., Chicago, IL, USA). To reduce the potential bias arising from some patients dying too early to receive initial therapy, the two patients who died within a month (30 days) of the start of initial therapy were excluded from the analysis.

## Results

The cohort consisted of 284 patients who were diagnosed as having stage IIIB or IV lung adenocarcinoma and had received initial therapy.

The clinical characteristics of the patients are summarized in Table 1. The median patient age prior to the start of initial therapy was 65 years (range, 23 to 87 years). The patients were predominantly younger than 70 years of age (81%), the ECOG PS was 0–2 in 93% of patients, and 91% of the patients had stage IV disease. While the lung adenocarcinoma patients with EGFR mutations were predominantly female (64%) and non-smokers (71%), those with wild-type EGFR were predominantly male (77%) and smokers (76%).

**Table 1:** Patient characteristics

**Characteristic**	**Mt + (n= 121)**		**Mt – (n= 163)**		**All (n= 284)**	
	**No.**	**%**	**No.**	**%**	**No.**	**%**
Age, years						
Median (range)	66 (32–87)		65 (23–83)		65 (23–87)	
< 70	97	80	134	82	231	81
≥ 70						
	24	20	29	18	53	19
Gender						
Male	43	36	125	77	168	59
Female	78	64	38	23	116	41
ECOG PS						
0-1	103	85	135	83	238	84
> 2	18	15	28	17	46	16
Smoking status						
Yes	50	41	124	76	174	61
No	71	59	39	24	110	39
Stage						
IIIB	6	5	19	12	25	9
IV	115	95	144	88	259	91
EGFR mutation						
Exon 19 deletion	59	49			59	21
Exon 21 L858R	57	47			57	20
Exon 18 G719X	5	4			5	2
Wild type			163	100	163	57
PCV						
Median (range)	1.6 (0.1-110.0)		2.3 (0.1-80.0)		2.0 (0.1-110.0)	
< 2.2 ng/ml	72	60	78	48	150	53
≥ 2.2 ng/ml	49	40	85	52	134	47
CEA						
Median (range)	8 (0.7-11942)		7 (0.5-14985)		7.4 (0.5-14985)	
< 5.0 ng/ml	45	37	63	39	108	38
≥ 5.0 ng/ml	76	63	100	61	176	62

EGFR: epidermal growth factor receptor, Mt+: mutant EGFR, Mt-: wild-type EGFR, PCV: pretreatment CYFRA 21–1 value.

Details about the first-line chemotherapy were available for 284 patients including both patient groups with wild-type (Mt-) and mutant EGFR (Mt+) groups (Table 2). About 40% of the EGFR mutation-positive patients received EGFR-TKIs as the initial treatment.

**Table 2:** Summary of initial treatment delivered among 284 patients

**EGFR mutation**	**Mt – (n= 163)**				**Mt + (n= 121)**			
	**IIIB**		**IV**		**IIIB**		**IV**	
	**(n= 19)**		**(n= 144)**		**(n= 6)**		**(n= 115)**	
	**No.**	**%**	**No.**	**%**	**No.**	**%**	**No.**	**%**
Treatment								
Platinum doublet	4	3	114	70	2	2	54	45
Monotherapy	0		30	18	0		11	9
EGFR-TKI	0		0		0		50	41
Chemoradiotherapy	15	9	0		4	3	0	
Specific regimens								
Cisplatin-pemetrexed	1		24	15	1		9	7
Carboplatin-paclitaxel	3		52	32	0		27	22
Carboplatin-paclitaxel+ bev	0		2		0		2	
Other platinum doublets	0		36	22	1		12	10
Gefitinib	0		0		0		41	34
Erlotinib	0		0		0		7	6
Docetaxel	0		16	10	0		3	
Vinorelbine	0		5		0		2	
Others	0		24	15	0		6	

Mt+: mutant EGFR, Mt-: wild-type EGFR, bev: bevacizumab.

Carboplatin-paclitaxel, the treatment of choice across both groups, was administered to half of the platinum doublet cohort in the Mt- patient group. Meanwhile, docetaxel was administered to half of the monotherapy cohort in the same patient group. However, cisplatin-pemetrexed was the most common regimen of second choice across both the Mt+ and Mt- groups.

**Figure 2:**
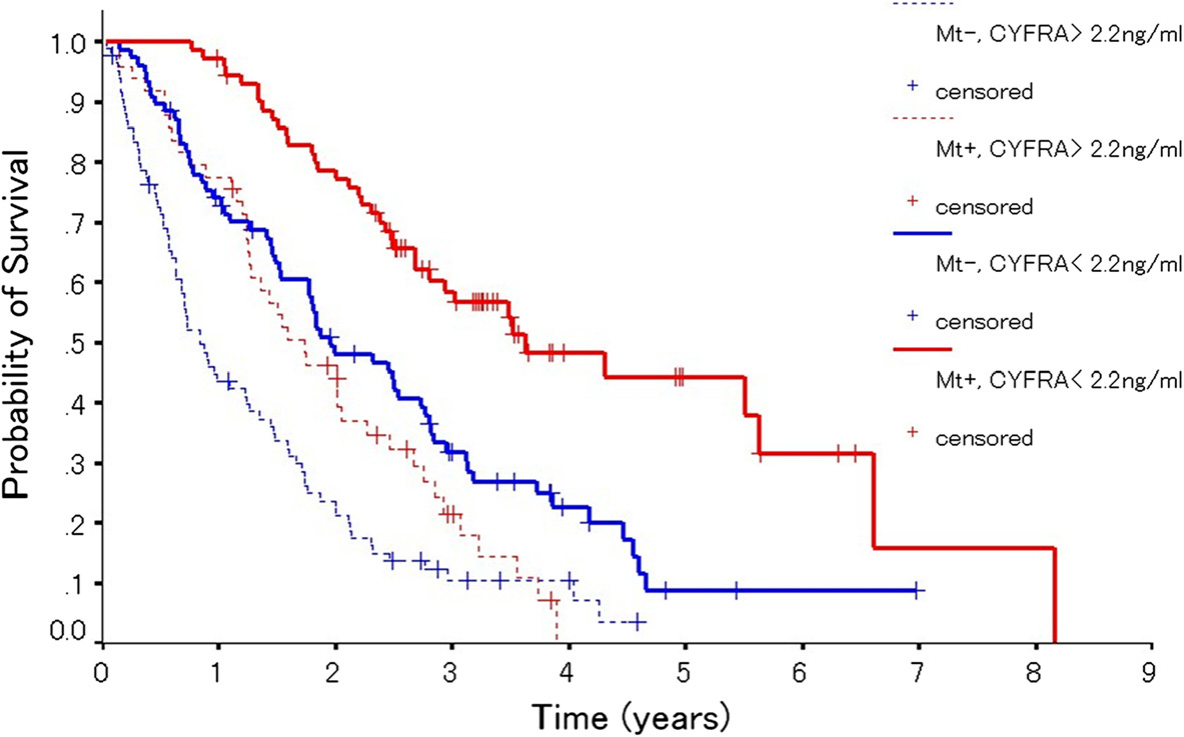
**Kaplan-Meier curves for overall survival in four groups, EGFR mutation status- stratified by PCV.** M+: mutant EGFR, M-: wild-type EGFR, PCV: pretreatment CYFRA 21–1 value.

The EGFR-TKI used for each treatment line in the Mt+ group is shown in Table 3. Forty-one (58%) patients received gefitinib, while 16 (22%) received erlotinib as first- or second-line treatment in the Mt+ group with PCV (<2.2 ng/ml). Thirty-seven (73%) patients received gefitinib, and 10 (20%) patients received erlotinib as first- or second-line treatment in the Mt+ group with PCV (≥2.2 ng/ml). Of the 121 patients in the Mt+ group, 27 did not receive gefitinib at any treatment-line stage of treatment; among these 27 patients, 19 received erlotinib (6 as first-line, 10 as second-line, 1 as third-line and 2 as further-line treatment). In the Mt+ group, a total of 113 patients (93%) received EGFR-TKIs, while 8 patients did not receive EGFR-TKIs at any stage of treatment. Furthermore, of the 160 patients in the Mt- group, 30 patients received EGFR-TKIs (11 as second-line, 7 as third-line, 6 as fourth-line, 3 as fifth-line, 1 as sixth-line, 1 as seventh-line, and 1 as eighth-line treatment). Fifty-three patients (18%) were still alive at the time of the analysis. The median follow-up period for determining the survival was 39.3 (range; 11.8-84.9) months after the start of initial therapy. The clinical variables identified by univariate analysis to be associated with significantly better survival (Table 4) included female gender (MST 32.4 months versus 20.1 months in males: p = .0086), no smoking history (33.4 months versus 20.1 months in smokers, p = .0012), ECOG PS (0–1) (29.5 months versus 7.9 months in those with a PS of 2–3, p < .0001), presence of EGFR mutation (39.2 months versus 17.8 months in patients without EGFR mutations, p < .0001), PCV < 2.2 ng/ml (38.6 months versus 15.0 months in those with PCV ≥ 2.2 ng/ml, p < .0001), serum CEA < 5.0 ng/ml (32.6 months versus 21.0 months in those with serum CEA ≥ 5.0 ng/ml, p = .036), start date of initial therapy before April 1, 2008 (34.1 months versus 19.3 months in the group that received the initial therapy after April 1, 2008, p = .003) and EGFR-TKI treatment (33.7 months versus 15.3 months in the group not treated with EGFR-TKIs, p < .0001). Multivariate analysis identified EGFR mutation positivity (HR 0.53; 95% CI: 0.34-0.84, p = .0069) and PCV < 2.2 ng/ml (HR 0.43; 95% CI: 0.31-0.59, p < .0001) as independent favorable prognostic factors. Another factor that was found to be an independent prognostic indicator of overall survival was the PS (Table 4). The overall survival rates of patients with advanced lung adenocarcinoma with/ without EGFR mutation are shown in Figure 2. Among the Mt+ patients, the prognosis was more favorable in the group with PCV < 2.2 ng/ml (n = 70) than in the group with PCV > 2.2 ng/ml (n = 48) (median survival time [MST]: 67.0 vs. 21.0 months, p < 0.0001). Among the patients with Mt- also, the prognosis was more favorable in the group with PCV < 2.2 ng/ml (n = 78) than in the group with PCV ≥ 2.2 ng/ml (n = 86) (MST: 24.1 vs. 10.2 months, p < 0.0001).

**Table 3:** Summary of EGFR-TKI delivered among EGFR mutation positive patients

	**EGFR mutation positive**							
	**Low PCV**				**High PCV**			
	**(< 2.2 ng/ml) (n= 72)**				**(≥ 2.2 ng/ml) (n= 49)**			
	**Gefitinib**		**Erlotinib**		**Gefitinib**		**Erlotinib**	
	**No.**	**%**	**No.**	**%**	**No.**	**%**	**No.**	**%**
First-line	20	28	5	7	23	47	2	4
Second-line	21	29	11	15	14	29	8	16
Third-line	9	12	6	8	3	6	4	8
Further-line	2	3	14	20	2	4	4	8
Unadministered	20	28	36	50	7	14	31	63

PCV: pretreatment CYFRA 21–1 value.

**Table 4:** Variables associated with overall survival among 284 patients

**Co-variable**	**No.**	**Univariate analysis**		**Multivariate analysis**			
		**MST (months)**	** P**	** Variate**	**OR**	**95% CI**	** P**
Age							
< 70	231	22.8					
> 70	53	24.3	0.625				
Gender							
Male	168	20.1					
Female	116	32.4	0.0086	Female	1.06	0.75-1.58	0.75
Smoking status							
Yes	174	20.1					
No	110	33.4	0.0012	No smoking status	0.84	0.52-1.24	0.39
ECOG PS							
0-1	238	29.5					
>2	46	7.9	<.0001	PS 0-1	0.34	0.24-0.50	<.0001
Stage							
IIIB	25	30.2					
IV	259	22.5	0.269				
EGFR mutation							
Mt (+)	121	39.2					
Mt (−)	163	17.8	<.0001	Mutant EGFR	0.53	0.34-0.84	0.0069
PCV							
< 2.2 ng/ml	150	38.6					
≥ 2.2 ng/ml	134	15.0	<.0001	< 2.2 ng/ml	0.43	0.31-0.59	<.0001
CEA							
< 5.0 ng/ml	108	32.6					
≥ 5.0 ng/ml	176	21.0	0.036	< 5.0 ng/ml	0.93	0.67-1.26	0.63
Start dates of IT							
Before 1/ 4/ 2008	79	34.1		After 1/ 4/ 2008			
After 1/ 4/ 2008	205	19.3	0.0030		0.73	0.50-1.15	0.07
EGFR-TKI treatment							
Yes	143	33.7					
No	141	15.3	<.0001	Yes	0.76	0.50-1.15	0.20

IT: initial therapy, PCV: pretreatment CYFRA 21–1 value, Mt(+): mutant EGFR, M(−): wild-type EGFR.

## Discussion

In the present study, we demonstrated PCV and EGFR mutation status as independent prognostic factors in untreated advanced lung adenocarcinoma patients. We also showed that PCV < 2.2 ng/ml was a predictor of a favorable outcome in both advanced lung adenocarcinoma patients with wild-type and mutant EGFR.

Serum CYFRA 21–1 has been reported as a prognostic factor in patients with a variety of cancer types, including resectable NSCLC [39,40], biliary tract cancer [41], urothelial cancer [42], head and neck cancer [43], esophageal cancer [44], and cervical cancer [45].

A meta-analysis of CYFRA 21–1 as a prognostic indicator in advanced NSCLC patients showed that the PCV may be a reliable prognostic factor [29], however, since non-adenocarcinoma accounted for 65% of the cases and squamous cell carcinoma for 50%, the role of serum CYFRA 21–1 as a prognostic indicator in the lung adenocarcinoma population remained unclear. Moreover, in a study of PCV as a prognostic indicator in advanced NSCLC patients in whom gefitinib was used as 3^rd^-line or later therapy, adenocarcinoma accounted for fewer than a half of the cases (47%) [30]. The EGFR mutation status was not included as a variable in the analysis, and the test population was small, consisting of only 50 patients.

Several factors may have contributed to identification of serum CYFRA 21–1 as a prognostic indicator in the advanced lung adenocarcinoma population in the present study. First, there could be a relationship between the serum levels of CYFRA 21–1 and the microfilament formation trend in the tumor cells [22]. CKs are the principal structural elements of intracellular microfilaments. Microfilaments have been shown to be heteropolymers formed from type I and type II keratins which form the cytoskeleton. Moreover, while the CKs (CKs 1, 2, 10/11), on which the degree of keratinization within tumors depends, are strongly expressed in well-differentiated squamous cell carcinomas, they are not detected in the serum. The possibility that they are preferentially removed by macrophages because of their poor solubility has been suggested as the reason for the failure to detect them in the serum [46]. By contrast, soluble CK19 is degraded by tumor lysis and tumor necrosis and released into the blood. Therefore, serum levels of CK19 may indicate the degree of cytoskeleton formation by microfilaments within the tumor cells. Second, there may also be a relationship between serum CYFRA 21–1 levels and the degree of tumor differentiation towards squamous epithelium. CKs with a relatively high molecular mass tend to be associated with differentiation into squamous cell carcinoma, while CKs with a relatively low molecular mass tend to be associated with differentiation into adenocarcinoma [47]. In a study in which monoclonal antibodies were used, the number of cells containing CK19 increased with decreasing degree of differentiation into squamous cell carcinoma, and the presence of intracellular CK19 was consistently demonstrated in pure lung adenocarcinomas [25]. On the other hand, a negative correlation between intracellular CK19 expression and serum CYFRA 21–1 levels has also been shown [24]. Increase in the serum level of CYFRA 21–1 may also be the result of a greater degree of degradation and release of intracellular CK19 into the serum with an increasing tendency towards differentiation into squamous cell carcinoma.

Because identical EGFR mutations have been seen in both the adenocarcinoma component and squamous cell carcinoma component in resected cases of adenosquamous carcinoma [48], it has been suggested that the two components may arise from a single clone [48,49]. Resected cases of adenosquamous carcinoma have been reported to account for 3% of all cases of NSCLC [50], and adenosquamous carcinoma patients have also been reported to have a poor prognosis [51]. The prognosis of patients in whom the tumor tissue consists of a mixture of mutant EGFR cells and wild-type EGFR cells has been reported to be inferior to that of patients with tumors consisting of only mutant EGFR cells, and intratumor heterogeneity has also been investigated [52]. On the other hand, there is a report suggesting that no intratumor heterogeneity of EGFR expression is found in mutant EGFR lung adenocarcinomas, and also that no disparity is found between the EGFR mutation status of the primary tumor and lymph node metastasis [53].

There are several limitations of the present study. The first is that it was a retrospective study conducted at a single institution, and the possibility of a selection bias is undeniable. The prognosis of patients who received initial therapy before April 1, 2008 was significantly superior to that of those who received their initial therapy after 2008. Because we started to perform EGFR mutation analysis in routine clinical practice from April 1, 2008, there is the possibility of a selection bias towards patients who received the initial therapy before April 1, 2008. This is one of the major limitations of our retrospective study. Some studies have reported that EGFR mutations may be a positive prognostic factor for survival in advanced NSCLC patients, regardless of EGFR-TKI therapy [54,55]. Also in the BR.21 trial, the median survival time was reported to be longer in patients with mutant EGFR as compared to that in patients with wild-type EGFR [56]. Although mutant EGFR patients not treated with EGFR-TKIs were found to be a confounding factor, we performed adjustment for the confounding factor using a Cox proportional hazards model. According to the univariate analysis, the date of start of the initial therapy (before April 1, 2008) was a favorable prognostic factor. However, PCV < 2.2 ng/ml, EGFR mutation positivity and PS 0–1 were found to be independent favorable prognostic factors after adjustment for the date of start of the initial therapy. In this study, while the MST (39.2 months) in the mutant EGFR group was not favorable as compared to previous reports [57], the mutant EGFR group with PCV < 2.2 ng/ml had a more favorable prognosis than that of the mutant EGFR group with PCV ≥ 2.2 ng/ml. The proportion of patients who received erlotinib was less in the group with PCV ≥ 2.2 ng/ml than in the group with PCV < 2.2 ng/ml, which could have influenced the more favorable prognosis in the group with PCV < 2.2 ng/ml than in the group with PCV ≥ 2.2 ng/ml. All of the patients with advanced lung adenocarcinoma in whom the diagnosis was made after April 1, 2008 were tested for EGFR mutations at the time of the diagnosis, whereas in the patients with other histological types of lung cancer, the testing was performed at the discretion of the attending physician. Second, the follow-up period was inadequate, especially in the mutant EGFR group with PCV < 2.2 ng/ml, and the censored cases were conspicuous. There was also a problem with the stage distribution (there were relatively few stage IIIB cases). Distant metastasis occurred in all of the stage IIIB cases in which local treatment had been performed, and all of the patients with disease recurrence were tested for EGFR mutations. Moreover, significant survival differences in stage IIIB/ IV were not found in the univariate analysis. Furthermore, the treatment regimens used in the stage IV cases were not standardized, with each of the attending physicians administering any of the various standard treatments used in routine clinical practice recommended by the guidelines of the Japan Lung Cancer Society.

In advanced lung adenocarcinoma, which may be considered as a generalized systemic disease, it may be particularly difficult to determine the characteristics of an entire heterogeneous tumor by tissue diagnosis alone based on examining just one part of the tumor. Based on the results of the present study, we propose that mutant EGFR patients with serum PCV < 2.2 ng/ml have a better prognosis than the mutant EGFR patients with higher PCV.

## Conclusions

The potential applications of PCV measurements might include identification of candidates in whom it might have some prognostic value. Furthermore, PCV might be regarded as a routine demographic variable having prognostic value in patients with advanced lung adenocarcinoma.

## Abbreviations

NSCLC: Non-small cell lung cancer; PCV: Pretreatment serum CYFRA 21–1 levels; pts: patients; EGFR: Epidermal growth factor receptor; Mt+: Mutant EGFR; Mt-: Wild-type; TKI: Tyrosine kinase inhibitor; CK: Cytokeratin; ECOG PS: Eastern Cooperative Oncology Group performance status; CLEIA: Chemiluminescence enzyme immunoassay; PCR: Polymerase chain reaction; IASLC: International Association of the Study of Lung Cancer; TNM: Tumor-node-metastasis.

## Competing interests

The authors have no competing interests to declare.

## Authors’ contributions

AO contributed to the drafting of this manuscript and data collection, and KM contributed to the study design and statistical analysis. TT, HA, TS, TT, HK, TN, HM, TN, ME, NY contributed to analysis of the data and interpretation of the findings. All authors have read and approved of the submission of the final manuscript.

## Pre-publication history

The pre-publication history for this paper can be accessed here:

http://www.biomedcentral.com/1471-2407/13/354/prepub

## References

[B1] World Health Organization Fact sheet N°297 (Cancer) 2012 http://www.who.int/mediacentre/factsheets/fs297/en/index.html23621790

[B2] American Cancer Society Lung Cancer (Non-small cell) 2012 1 68 http://www.cancer.org/cancer/lungcancer-non-smallcell/detailedguide/non-small-cell-lung-cancer-what-is-non-small-cell-lung-cancer

[B3] Scagliotti GV Parikh P von Pawel J Biesma B Vansteenkiste J Manegold C Serwatowski P Gatzemeier U Digumarti R Zukin M Lee JS Mellemgaard A Park K Patil S Rolski J Goksel T de Marinis F Simms L Sugarman KP Gandara D Phase III study comparing cisplatin plus gemcitabine with cisplatin plus pemetrexed in chemotherapy-naive patients with advanced-stage non-small-cell lung cancer J Clin Oncol 2008 26 3543 3551 10.1200/JCO.2007.15.0375 18506025

[B4] Sandler A Gray R Perry MC Brahmer J Schiller JH Dowlati A Lilenbaum R Johnson DH Paclitaxel-carboplatin alone or with bevacizumab for non-small-cell lung cancer N Engl J Med 2006 355 2542 2550 10.1056/NEJMoa061884 17167137

[B5] Niho S Kunitoh H Nokihara H Horai T Ichinose Y Hida T Yamamoto N Kawahara M Shinkai T Nakagawa K Matsui K Negoro S Yokoyama A Kudoh S Kiura K Mori K Okamoto H Sakai H Takeda K Yokota S Saijo N Fukuoka M Randomized phase II study of first-line carboplatin-paclitaxel with or without bevacizumab in Japanese patients with advanced non-squamous non-small-cell lung cancer Lung Cancer 2012 76 363 367 10.1016/j.lungcan.2011.12.005 22244743

[B6] Lynch TJ Bell DW Sordella R Gurubhagavatula S Okimoto RA Brannigan BW Harris PL Haserlat SM Supko JG Haluska FG Louis DN Christiani DC Settleman J Haber DA Activating mutations in the epidermal growth factor receptor underlying responsiveness of non-small-cell lung cancer to gefitinib N Engl J Med 2004 350 2129 2139 10.1056/NEJMoa040938 15118073

[B7] Paez JG Jänne PA Lee JC Tracy S Greulich H Gabriel S Herman P Kaye FJ Lindeman N Boggon TJ Naoki K Sasaki H Fujii Y Eck MJ Sellers WR Johnson BE Meyerson M EGFR mutations in lung cancer: correlation with clinical response to gefitinib therapy Science 2004 304 1497 1500 10.1126/science.1099314 15118125

[B8] Mok TS Wu YL Thongprasert S Yang CH Chu DT Saijo N Sunpaweravong P Han B Margono B Ichinose Y Nishiwaki Y Ohe Y Yang JJ Chewaskulyong B Jiang H Duffield EL Watkins CL Armour AA Fukuoka M Gefitinib or carboplatin-paclitaxel in pulmonary adenocarcinoma N Engl J Med 2009 361 947 957 10.1056/NEJMoa0810699 19692680

[B9] Maemondo M Inoue A Kobayashi K Sugawara S Oizumi S Isobe H Gemma A Harada M Yoshizawa H Kinoshita I Fujita Y Okinaga S Hirano H Yoshimori K Harada T Ogura T Ando M Miyazawa H Tanaka T Saijo Y Hagiwara K Morita S Nukiwa T Gefitinib or chemotherapy for non-small-cell lung cancer with mutated EGFR N Engl J Med 2010 362 2380 2388 10.1056/NEJMoa0909530 20573926

[B10] Rosell R Carcereny E Gervais R Vergnenegre A Massuti B Felip E Palmero R Garcia-Gomez R Pallares C Sanchez JM Porta R Cobo M Garrido P Longo F Moran T Insa A De Marinis F Corre R Bover I Illiano A Dansin E de Castro J Milella M Reguart N Altavilla G Jimenez U Provencio M Moreno MA Terrasa J Muñoz-Langa J Erlotinib versus standard chemotherapy as first-line treatment for European patients with advanced EGFR mutation-positive non-small-cell lung cancer (EURTAC): a multicentre, open-label, randomised phase 3 trial Lancet Oncol 2012 13 239 246 10.1016/S1470-2045(11)70393-X 22285168

[B11] Mitsudomi T Morita S Yatabe Y Negoro S Okamoto I Tsurutani J Seto T Satouchi M Tada H Hirashima T Asami K Katakami N Takada M Yoshioka H Shibata K Kudoh S Shimizu E Saito H Toyooka S Nakagawa K Fukuoka M Gefitinib versus cisplatin plus docetaxel in patients with non-small-cell lung cancer harbouring mutations of the epidermal growth factor receptor (WJTOG3405): an open label, randomised phase 3 trial Lancet Oncol 2010 11 121 128 10.1016/S1470-2045(09)70364-X 20022809

[B12] Huang SF Liu HP Li LH Ku YC Fu YN Tsai HY Chen YT Lin YF Chang WC Kuo HP Wu YC Chen YR Tsai SF High frequency of epidermal growth factor receptor mutations with complex patterns in non-small cell lung cancers related to gefitinib responsiveness in Taiwan Clin Cancer Res 2004 10 8195 8203 10.1158/1078-0432.CCR-04-1245 15623594

[B13] Kosaka T Yatabe Y Endoh H Kuwano H Takahashi T Mitsudomi T Mutations of the epidermal growth factor receptor gene in lung cancer: biological and clinical implications Cancer Res 2004 64 8919 8923 10.1158/0008-5472.CAN-04-2818 15604253

[B14] Shigematsu H Lin L Takahashi T Nomura M Suzuki M Wistuba II Fong KM Lee H Toyooka S Shimizu N Fujisawa T Feng Z Roth JA Herz J Minna JD Gazdar AF Clinical and biological features associated with epidermal growth factor receptor gene mutations in lung cancers J Natl Cancer Inst 2005 97 339 346 10.1093/jnci/dji055 15741570

[B15] Chou TY Chiu CH Li LH Hsiao CY Tzen CY Chang KT Chen YM Perng RP Tsai SF Tsai CM Mutation in the tyrosine kinase domain of epidermal growth factor receptor is a predictive and prognostic factor for gefitinib treatment in patients with non-small cell lung cancer Clin Cancer Res 2005 11 3750 3757 10.1158/1078-0432.CCR-04-1981 15897572

[B16] Park SH Ha SY Lee JI Lee H Sim H Kim YS Hong J Park J Cho EK Shin DB Lee JH Epidermal growth factor receptor mutations and the clinical outcome in male smokers with squamous cell carcinoma of lung J Krean Med Sci 2009 24 448 452 10.3346/jkms.2009.24.3.448 PMC2698191 19543508

[B17] Miyamae Y Shimizu K Hirato J Araki T Tanaka K Ogawa H Kakegawa S Sugano M Nakano T Mitani Y Kaira K Takeyoshi I Significance of epidermal growth factor receptor gene mutations in squamous cell lung carcinoma Oncol Rep 2011 25 921 928 21318227 10.3892/or.2011.1182

[B18] Ohtsuka K Ohnishi H Fujiwara M Kishino T Matsushima S Furuyashiki G Takei H Koshiishi Y Goya T Watanabe T Abnormalities of epidermal growth factor receptor in lung squamous-cell carcinomas, adenosquamous carcinomas, and large-cell carcinomas: tyrosine kinase domain mutations are not rare in tumors with an adenocarcinoma component Cancer 2007 109 741 750 10.1002/cncr.22476 17238183

[B19] Rekhtman N Paik PK Arcila ME Tafe LJ Oxnard GR Moreira AL Travis WD Zakowski MF Kris MG Ladanyi M Clarifying the spectrum of driver oncogene mutations in biomarker-verified squamous carcinoma of lung: lack of EGFR/KRAS and presence of PIK3CA/AKT1 mutations Clin Cancer Res 2012 18 1167 1176 10.1158/1078-0432.CCR-11-2109 22228640 PMC3487403

[B20] Moll R Franke WW Schiller DL Geiger B Krepler R The catalog of human cytokeratins: patterns of expression in normal epithelia, tumors and cultured cells Cell 1982 31 11 24 10.1016/0092-8674(82)90400-7 6186379

[B21] Moll R Löwe A Laufer J Franke WW Cytokeratin 20 in human carcinomas. A new histodiagnostic marker detected by monoclonal antibodies Am J Pathol 1992 140 427 447 1371204 PMC1886432

[B22] Hatzfeld M Franke WW Pair formation and promiscuity of cytokeratins: formation in vitro of heterotypic complexes and intermediate-sized filaments by homologous and heterologous recombinations of purified polypeptides J Cell Biol 1985 101 1826 1841 10.1083/jcb.101.5.1826 2414304 PMC2113979

[B23] Rastel D Ramaioli A Cornillie F Thirion B CYFRA 21–1, a sensitive and specific new tumour marker for squamous cell lung cancer. Report of the first European multicentre evaluation. CYFRA 21–1 Multicentre Study Group Eur J Cancer 1994 30A 601 606 7521651 10.1016/0959-8049(94)90528-2

[B24] Kosacka M Jankowska R Comparison of cytokeratin 19 expression in tumor tissue and serum CYFRA 21–1 levels in non-small cell lung cancer Pol Arch Med Wewn 2009 119 33 37 19341176

[B25] Broers JL Ramaekers FC Rot MK Oostendorp T Huysmans A van Muijen GN Wagenaar SS Vooijs GP Cytokeratins in different types of human lung cancer as monitored by chain-specific monoclonal antibodies Cancer Res 1988 48 3221 3229 2452687

[B26] Dohmoto K Hojo S Fujita J Yang Y Ueda Y Bandoh S Yamaji Y Ohtsuki Y Dobashi N Ishida T Takahara J The role of caspase 3 in producing cytokeratin 19 fragment (CYFRA21-1) in human lung cancer cell lines Int J Cancer 2001 91 468 473 10.1002/1097-0215(200002)9999:9999<::AID-IJC1082>3.0.CO;2-T 11251967

[B27] Satoh H Ishikawa H Fujiwara M Yamashita YT Ohtsuka M Ogata T Hasegawa S Kamma H Production of cytokeratin 19 fragment by human squamous lung cancer cell lines Am J Respir Cell Mol Biol 1997 16 597 604 10.1165/ajrcmb.16.5.9160842 9160842

[B28] Pujol JL Grenier J Daurès JP Daver A Pujol H Michel FB Serum fragment of cytokeratin subunit 19 measured by CYFRA 21–1 immunoradiometric assay as a marker of lung cancer Cancer Res 1993 53 61 66 7677981

[B29] Pujol JL Molinier O Ebert W Daurès JP Barlesi F Buccheri G Paesmans M Quoix E Moro-Sibilot D Szturmowicz M Bréchot JM Muley T Grenier J CYFRA 21–1 is a prognostic determinant in non-small-cell lung cancer: results of a meta-analysis in 2063 patients Br J Cancer 2004 90 2097 2105 15150567 10.1038/sj.bjc.6601851 PMC2409493

[B30] Barlési F Tchouhadjian C Doddoli C Torre JP Astoul P Kleisbauer JP CYFRA 21–1 level predicts survival in non-small-cell lung cancer patients receiving gefitinib as third-line therapy Br J Cancer 2005 92 13 14 10.1038/sj.bjc.6602296 15597098 PMC2361739

[B31] Park SY Lee JG Kim J Park Y Lee SK Bae MK Lee CY Kim DJ Chung KY Preoperative serum CYFRA 21–1 level as a prognostic factor in surgically treated adenocarcinoma of lung Lnug Cancer 2013 79 156 160 10.1016/j.lungcan.2012.11.006 23206831

[B32] Jung M Kim SH Hong S Kang YA Kim SK Chang J Rha SY Kim JH Kim DJ Cho BC Prognostic and predictive value of carcinoembryonic antigen and cytokeratin-19 fragments levels in advanced non-small cell lung cancer patients treated with gefitinib or erlotinib Yonsei Med J 2012 53 931 939 10.3349/ymj.2012.53.5.931 22869475 PMC3423836

[B33] Edelman MJ Hodgson L Rosenblatt PY Christenson RH Vokes EE Wang X Kratzke R CYFRA 21–1 as a prognostic and predictive marker in advanced non-small-cell lung cancer in a prospective trial: CALGB 150304 J Thorac Oncol 2012 7 649 654 10.1097/JTO.0b013e31824a8db0 22425913 PMC5541770

[B34] Jung M Kim SH Lee YJ Hong S Kang YA Kim SK Chang J Rha SY Kim JH Kim DJ Cho BC Prognostic and predictive value of CEA and CYFRA 21–1 levels in advanced non-small cell lung cancer patients treated with gefitinib or erlotinib Exp Ther Med 2011 2 685 693 22977560 10.3892/etm.2011.273 PMC3440747

[B35] Travis WD Brambilla E Muller-Hermelink HK Harris CC World Health Organization Classification of Tumors. Pathology and Genetics of Tumors of the Lung, Pleura, Thymus and Heart 2004 Lyon: IARC Press

[B36] Kawai T Ohkubo A Hasegawa S Kuriyama T Kato H Fukuoka M Ohkawa J Yotsumoto H Sugama Y Kawate N Takada M Tatsumi K Satoh H Kitamura S Reference value and cutoff value, diagnostic specificity, sensitivity by EIA measuring method of new marker of lung cancer CYFRA J Clin Lab Inst Reag 1993 16 1232 1238

[B37] Kuroda M Aizu M Shimazu C Miyazawa Y Evaluation of CYFRA21-1 measurement by fully automated chemiluminescent immunoassay system “Lumipulse Presto” J Clin Lab Inst Reag 2006 29 597 602

[B38] Goldstraw P Crowley J Chansky K Giroux DJ Groome PA Rami-Porta R Postmus PE Rusch V Sobin L The IASLC Lung Cancer Staging Project: proposals for the revision of the TNM stage groupings in the forthcoming (seventh) edition of the TNM Classification of malignant tumours J Thorac Oncol 2007 2 706 714 10.1097/JTO.0b013e31812f3c1a 17762336

[B39] Reinmuth N Brandt B Semik M Kunze WP Achatzy R Scheld HH Broermann P Berdel WE Macha HN Thomas M Prognostic impact of Cyfra21-1 and other serum markers in completely resected non-small cell lung cancer Lung Cancer 2002 36 265 270 10.1016/S0169-5002(02)00009-0 12009236

[B40] Hanagiri T Sugaya M Takenaka M Oka S Baba T Shigematsu Y Nagata Y Shimokawa H Uramoto H Takenoyama M Yasumoto K Tanaka F Preoperative CYFRA 21–1 and CEA as prognostic factors in patients with stage I non-small cell lung cancer Lung Cancer 2011 74 112 117 10.1016/j.lungcan.2011.02.001 21397974

[B41] Chapman MH Sandanayake NS Andreola F Dhar DK Webster GJ Dooley JS Pereira SP Circulating CYFRA 21–1 is a Specific Diagnostic and Prognostic Biomarker in Biliary Tract Cancer J Clin Exp Hepatol 2011 1 6 12 22228935 10.1016/S0973-6883(11)60110-2 PMC3252025

[B42] Suyama T Nakajima K Kanbe S Tanaka N Hara H Ishii N Prognostic significance of preoperative serum CYFRA 21–1 in patients with upper urinary tract urothelial carcinoma Int J Urol 2011 18 43 47 10.1111/j.1442-2042.2010.02671.x 21070384

[B43] Doweck I Barak M Uri N Greenberg E The prognostic value of the tumour marker Cyfra 21–1 in carcinoma of head and neck and its role in early detection of recurrent disease Br J Cancer 2000 83 1696 1701 10.1054/bjoc.2000.1502 11104568 PMC2363457

[B44] Shimada H Nabeya Y Okazumi S Matsubara H Miyazawa Y Shiratori T Hayashi H Gunji Y Ochiai T Prognostic significance of CYFRA 21–1 in patients with esophageal squamous cell carcinoma J Am Coll Surg 2003 196 573 578 10.1016/S1072-7515(02)01905-1 12691934

[B45] Bonfrer JM Gaarenstroom KN Kenter GG Korse CM Hart AA Gallee MP Helmerhorst TJ Kenemans P Prognostic significance of serum fragments of cytokeratin 19 measured by Cyfra 21–1 in cervical cancer Gynecol Oncol 1994 55 371 375 10.1006/gyno.1994.1309 7530676

[B46] Miédougé M Devys A Simon M Rouzaud P Salama G Reyre J Pujazon M Carles P Serre G High levels of cytokeratin 19 fragments but no evidence of cytokeratins 1, 2, 10/11, 14 or filaggrin in the serum of squamous cell lung carcinoma patients Tumour Biol 2001 22 19 26 10.1159/000030151 11054023

[B47] Ramaekers F Huysmans A Moesker O Kant A Jap P Herman C Vooijs P Monoclonal antibody to keratin filaments, specific for glandular epithelia and their tumors. Use in surgical pathology Lab Invest 1983 49 353 361 6193333

[B48] Kang SM Kang HJ Shin JH Kim H Shin DH Kim SK Kim JH Chung KY Kim SK Chang J Identical epidermal growth factor receptor mutations in adenocarcinomatous and squamous cell carcinomatous components of adenosquamous carcinoma of the lung Cancer 2007 109 581 587 10.1002/cncr.22413 17186532

[B49] Niho S Yokose T Kodama T Nishiwaki Y Mukai K Clonal analysis of adenosquamous carcinoma of the lung Jpn J Cancer Res 1999 90 1244 1247 10.1111/j.1349-7006.1999.tb00703.x 10622536 PMC5926013

[B50] Goya T Asamura H Yoshimura H Kato H Shimokata K Tsuchiya R Sohara Y Miya T Miyaoka E Prognosis of 6644 resected non-small cell lung cancers in Japan: a Japanese lung cancer registry study Lung Cancer 2005 50 227 234 10.1016/j.lungcan.2005.05.021 16061304

[B51] Gawrychowski J Bruliński K Malinowski E Papla B Prognosis and survival after radical resection of primary adenosquamous lung carcinoma Eur J Cardiothorac Surg 2005 27 686 692 10.1016/j.ejcts.2004.12.030 15784375

[B52] Taniguchi K Okami J Kodama K Higashiyama M Kato K Intratumor heterogeneity of epidermal growth factor receptor mutations in lung cancer and its correlation to the response to gefitinib Cancer Sci 2008 99 929 935 10.1111/j.1349-7006.2008.00782.x 18325048 PMC11158886

[B53] Yatabe Y Matsuo K Mitsudomi T Heterogeneous distribution of EGFR mutations is extremely rare in lung adenocarcinoma J Clin Oncol 2011 29 2972 2977 10.1200/JCO.2010.33.3906 21730270

[B54] Eberhard DA Johnson BE Amler LC Goddard AD Heldens SL Herbst RS Ince WL Jänne PA Januario T Johnson DH Klein P Miller VA Ostland MA Ramies DA Sebisanovic D Stinson JA Zhang YR Seshagiri S Hillan KJ Mutations in the epidermal growth factor receptor and in KRAS are predictive and prognostic indicators in patients with non-small-cell lung cancer treated with chemotherapy alone and in combination with erlotinib J Clin Oncol 2005 23 5900 5909 10.1200/JCO.2005.02.857 16043828

[B55] Bell DW Lynch TJ Haserlat SM Harris PL Okimoto RA Brannigan BW Sgroi DC Muir B Riemenschneider MJ Iacona RB Krebs AD Johnson DH Giaccone G Herbst RS Manegold C Fukuoka M Kris MG Baselga J Ochs JS Haber DA Epidermal growth factor receptor mutations and gene amplification in non-small-cell lung cancer: molecular analysis of the IDEAL/INTACT gefitinib trials J Clin Oncol 2005 23 8081 8092 10.1200/JCO.2005.02.7078 16204011

[B56] Zhu CQ da Cunha Santos G Ding K Sakurada A Cutz JC Liu N Zhang T Marrano P Whitehead M Squire JA Kamel-Reid S Seymour L Shepherd FA Tsao MS National Cancer Institute of Canada Clinical Trials Group Study BR.21 Role of KRAS and EGFR as biomarkers of response to erlotinib in National Cancer Institute of Canada Clinical Trials Group Study BR.21 J Clin Oncol 2008 26 4268 4275 10.1200/JCO.2007.14.8924 18626007

[B57] Mitsudomi T Morita S Yatabe Y Negoro S Okamoto I Seto T Satouchi M Tada H Hirashima T Asami K Katakami N Takada M Yoshioka H Shibata K Kudoh S Shimizu E Saito H Toyooka S Nakagawa K Fukuoka M Updated overall survival results of WJTOG 3405, a randomized phase III trial comparing gefitinib (G) with cisplatin plus docetaxel (CD) as the first-line treatment for patients with non-small cell lung cancer harboring mutations of the epidermal growth factor receptor (EGFR) Proc Am Soc Clin Oncol 2012 30 7521

